# Bibliometric Analysis of Neonatal Sepsis Research in Sub-Saharan Africa (2000-2025): Trends, Funding Patterns, and Collaboration Networks

**DOI:** 10.7759/cureus.113561

**Published:** 2026-07-28

**Authors:** Funso A Olagunju, Sunday C Adeyemo, Kehinde Awodele, Abisola O Omoboyeje, Temitope O Ayeni, Efeturi Agelebe, Opeyemi Q Asafa, Ganiyu A Oyeniyi, Eniola D Olabode, Ayodele Ajayi

**Affiliations:** 1 Department of Paediatrics and Child Health, Osun State University, Osogbo, NGA; 2 Department of Health and Social Care, QA Higher Education, London, GBR; 3 Department of Public Health Research, University of Wolverhampton, Wolverhampton, GBR; 4 Department of Health and Biomedical Sciences, Institut Supérieur de Santé, Niamey, NER; 5 Department of Obstetrics and Gynaecology, Osun State University, Osogbo, NGA; 6 Department of Paediatrics and Child Health, Bowen University, Iwo, NGA; 7 Department of Surgery, Osun State University, Osogbo, NGA; 8 Department of Community Medicine, Institut Supérieur de Santé, Niamey, NER; 9 Department of Nursing, Pear Tree Court Care Home - Care UK, Waterlooville, GBR; 10 Department of Psychiatry, Afe Babalola University Teaching Hospital, Ado-Ekiti, NGA

**Keywords:** bibliometric analysis, global health collaboration, neonatal sepsis, research equity, research funding, sub-saharan africa

## Abstract

Neonatal sepsis accounts for a substantial proportion of newborn mortality in Sub-Saharan Africa (SSA), yet the region remains under-represented in global research output. While bibliometric analyses of neonatal sepsis exist globally, none have mapped 25-year trends, funding sources, and collaboration networks specific to SSA. Understanding these patterns is critical to address research inequity and guide evidence-based investment. We conducted a bibliometric analysis of publications on neonatal sepsis in SSA indexed in Scopus and Web of Science Core Collection from 2000 to 2025. A comprehensive search string captured neonatal sepsis terms and all 46 SSA countries. After deduplication and screening per PRISMA 2020 guidelines, data were analyzed using the Bibliometrix R package. Outcomes included publication trends, geographic productivity, funding sources, co-authorship networks, and keyword co-occurrence to identify research themes and gaps. A total of 1,247 publications were included. Annual output increased from 12 articles in 2000 to a peak of 106 in 2024, followed by a slight decline to 98 in 2025, with an average annual growth rate of 8.2% (95% CI: 7.1-9.3%). South Africa (28.3%), Nigeria (15.7%), and Kenya (10.2%) contributed 54.2% of total publications. The top institutions were University of Cape Town (n = 94), Makerere University (n = 67), and University of Nairobi (n = 58). Funding analysis showed that 82.3% of funded studies were supported by high-income country agencies, primarily National Institutes of Health (NIH) (15.6%), Wellcome Trust (12.3%), and Bill & Melinda Gates Foundation (9.8%), while African national funders accounted for <6.5%. Country collaboration networks revealed strong North-South links but limited South-South collaboration, with a multiple-country publication ratio of 0.34. Keyword analysis identified microbiology (18.4%) and antibiotic resistance (14.2%) as dominant themes, while implementation science (2.1%) and health systems research (1.8%) remained underrepresented. Despite a 25-year increase in output, neonatal sepsis research in SSA remains disproportionately low relative to disease burden and heavily dependent on external funding. Strengthening African-led funding mechanisms and fostering South-South collaborations are essential to build sustainable research capacity and align research priorities with local mortality burden.

## Introduction and background

Neonatal sepsis, a life-threatening systemic blood infection occurring in newborns within the first 28 days of life, remains a leading cause of newborn mortality worldwide, with Sub-Saharan Africa (SSA) bearing the highest burden. Approximately 2.5 million neonatal deaths occur annually, with sepsis contributing to 15-25% of these fatalities [1,2]. The incidence of neonatal sepsis in SSA ranges from 11 to 45 per 1,000 live births, substantially higher than in high-income countries (HICs) where rates are one to three per 1,000 live births [3,4]. In SSA, this vulnerability is exacerbated by unsterile delivery environments, limited access to clean cord care, delays in seeking care, and weak health systems that struggle to provide timely diagnosis and appropriate antibiotics - factors that transform a treatable infection into a fatal one. This disparity is compounded by limited diagnostic capacity, antimicrobial resistance, and weak health systems in many SSA settings [5,6].

To understand why research output matters, it helps to consider a simple question: How do we know what research is being done, where it is happening, and who is funding it? Traditional literature reviews summarize what studies have found, but they cannot tell us whether the volume of research matches the scale of the problem, whether funding is equitably distributed, or whether researchers from different regions are working together. Bibliometric analysis, the systematic examination of publication patterns, citations, and collaborations, provides this missing picture. It reveals which countries and institutions are most active, which funders are supporting the work, and whether partnerships are truly collaborative or merely extractive.

Despite bearing the highest global burden of neonatal sepsis mortality, SSA remains underrepresented in research output, with disproportionately low bibliometric visibility [7,8]. Existing bibliometric analyses cover global trends in neonatal sepsis research but do not provide a region-specific, longitudinal assessment of publication trends, funding sources, or collaboration networks for SSA [9,10]. When we refer to "North-South" collaboration, we mean partnerships between researchers in HICs (primarily in Europe and North America) and those in low- and middle-income countries like those in SSA. "South-South" collaboration refers to partnerships among researchers within low- and middle-income countries themselves, for example, between a university in Kenya and one in Nigeria. Understanding the balance between these types of partnerships is important because it reveals whether research capacity is being built locally or whether it remains dependent on external actors. This gap in the literature limits the ability of policymakers and funders to make evidence-based decisions about research prioritization and capacity building in the region [11].

Regional bibliometric analyses have been conducted for other high-burden regions. For example, studies from South Asia have mapped neonatal sepsis research trends in India, Bangladesh, and Pakistan, revealing similar patterns of low output relative to disease burden but with stronger South-South collaboration networks within the region [12,13]. In Latin America, bibliometric analyses have highlighted the dominance of Brazil and Mexico in regional output and the critical role of Pan American Health Organization funding [14]. However, to our knowledge, no comparable 25-year analysis has been conducted for SSA that simultaneously examines publication trends, funding sources, and collaboration networks in a single integrated framework.

Bibliometric analysis offers a powerful tool to systematically evaluate research activity, identify funding patterns, and map collaboration networks within a specific field [15]. By analyzing publication metadata, researchers can quantify productivity, visualize scientific communities, and identify research gaps that may not be apparent through traditional literature reviews [16]. In the context of neonatal sepsis in SSA, such analysis can reveal the extent to which research investment aligns with disease burden and identify opportunities for more equitable research partnerships.

By examining these patterns, we can answer the following critical questions: Is research investment proportional to the burden of disease? Are funders from within Africa stepping up, or does the region remain dependent on external donors? Are researchers from different African countries learning from each other, or are they primarily connected through partners in Europe and North America? This study addresses these questions through a 25-year bibliometric analysis from 2000 to 2025, aiming to fill this gap and guide future research prioritization, funding allocation, and collaborative partnerships in the region. Specifically, we aimed to map publication trends, geographic productivity, funding sources, collaboration networks, and research themes to provide actionable insights for stakeholders committed to reducing neonatal sepsis mortality in SSA.

## Review

Method

Search Strategy

Scopus and Web of Science (WoS) Core Collection were searched for publications on neonatal sepsis in SSA published between January 1, 2000, and December 31, 2025. We selected Scopus and Web of Science Core Collection because they provide structured affiliation and funding metadata (AU, AU_CO, FU, FO) essential for collaboration network and funding source analyses [17,18]. Unlike PubMed/MEDLINE, which lacks these fields, Scopus and WoS are the preferred sources for large-scale bibliometric mapping [19]. The exclusion of PubMed was therefore intentional and methodologically justified.

Inclusion criteria: Studies focused on neonatal sepsis, early-onset sepsis, late-onset sepsis, neonatal infection, or neonatal bacteremia. The studies must include SSA context. Studies conducted in, about, or with data from ≥1 country in SSA. Original research, reviews, meta-analyses, editorials, letters, or conference papers with full data published in English between 1 January 2000 and 31 December 2025, and studies indexed in Scopus and/or Web of Science Core Collection.

Exclusion criteria: Global studies with no SSA-specific data or <5% SSA sample; non-human studies or animal models; in vitro studies without clinical relevance; studies only on maternal sepsis; abstract-only, retracted articles; and books.

Search Strings

The search strategy for Scopus Advanced Search (verbatim syntax) was as follows: TITLE-ABS-KEY (("neonatal sepsis" OR "neonatal septicemia" OR "neonatal infection*" OR "early-onset sepsis" OR "late-onset sepsis" OR "newborn sepsis" OR "neonatal bacteremia") AND ("Sub-Saharan Africa" OR "Sub Saharan Africa" OR Angola OR Benin OR Botswana OR "Burkina Faso" OR Burundi OR "Cabo Verde" OR Cameroon OR "Central African Republic" OR Chad OR Comoros OR Congo OR "Cote d'Ivoire" OR "Ivory Coast" OR "Democratic Republic of the Congo" OR Djibouti OR "Equatorial Guinea" OR Eritrea OR Eswatini OR Ethiopia OR Gabon OR Gambia OR Ghana OR Guinea OR "Guinea-Bissau" OR Kenya OR Lesotho OR Liberia OR Madagascar OR Malawi OR Mali OR Mauritania OR Mauritius OR Mozambique OR Namibia OR Niger OR Nigeria OR Rwanda OR "Sao Tome and Principe" OR Senegal OR Seychelles OR "Sierra Leone" OR Somalia OR "South Africa" OR "South Sudan" OR Tanzania OR Togo OR Uganda OR Zambia OR Zimbabwe)) AND PUBYEAR > 1999 AND PUBYEAR < 2026 AND LANGUAGE (English) AND DOCTYPE (ar OR re OR cp OR ed OR le).

The search strategy for Web of Science Core Collection (verbatim syntax) was as follows: TS=(("neonatal sepsis" OR "neonatal septicemia" OR "neonatal infection*" OR "early-onset sepsis" OR "late-onset sepsis" OR "newborn sepsis" OR "neonatal bacteremia") AND ("Sub-Saharan Africa" OR Angola OR Benin OR Botswana OR "Burkina Faso" OR Burundi OR "Cabo Verde" OR Cameroon OR "Central African Republic" OR Chad OR Comoros OR Congo OR "Cote d'Ivoire" OR "Ivory Coast" OR "Democratic Republic of the Congo" OR Djibouti OR "Equatorial Guinea" OR Eritrea OR Eswatini OR Ethiopia OR Gabon OR Gambia OR Ghana OR Guinea OR "Guinea-Bissau" OR Kenya OR Lesotho OR Liberia OR Madagascar OR Malawi OR Mali OR Mauritania OR Mauritius OR Mozambique OR Namibia OR Niger OR Nigeria OR Rwanda OR "Sao Tome and Principe" OR Senegal OR Seychelles OR "Sierra Leone" OR Somalia OR "South Africa" OR "South Sudan" OR Tanzania OR Togo OR Uganda OR Zambia OR Zimbabwe)) AND PY=2000-2025 AND DT=(Article OR Review OR Proceedings Paper OR Editorial Material OR Letter) AND LA=(English).

Screening Process

The PRISMA 2020 guidelines for systematic reviews were followed to ensure transparency in our screening process [20]. Scopus and Web of Science results were exported to .bib and .csv formats. Duplicates were removed using Rayyan and Zotero reference management software. Titles and abstracts were screened for SSA relevance by two independent reviewers (Author 1 and Author 2), with discrepancies resolved by a third reviewer (Author 3). Full-text screening was performed to ensure data completeness and eligibility verification. The final set was exported to Bibliometrix R for analysis. Inter-rater reliability was assessed using Cohen's kappa coefficient (κ = 0.87, 95% CI: 0.82-0.92), indicating strong agreement between reviewers [21]. Disagreements (n = 67, 4.5% of screened records) were primarily related to whether a study met the ≥5% SSA sample threshold and were resolved through discussion and, when necessary, adjudication by the third reviewer.

A total of 4,770 records were identified from Scopus (n = 2,847) and Web of Science (n = 1,923). After removing duplicates (n = 1,646), 3,124 records were screened. Following title/abstract screening, 1,632 records were excluded (987 not SSA focus, 423 not neonatal sepsis, and 222 animal/in-vitro studies). Full-text assessment of 1,492 records led to the exclusion of 245 studies (134 no SSA data, 78 no full text, and 33 retracted).

Data Analysis

Bibliometric analysis was conducted using the Bibliometrix R package (version 4.1.4) [22,23]. Annual publication counts, average annual growth rate, and citation analysis were performed using descriptive statistics. Country and institutional productivity was ranked by publication count and total citations. The multiple country publication (MCP) ratio was calculated to measure international collaboration.

Collaboration networks: Co-authorship networks at the country and institutional levels were constructed using the biblioNetwork function in Bibliometrix with analysis = "collaboration" and network = "countries" (or "institutions").

Funding agencies were extracted from the "FU" (Scopus) and "FO" (Web of Science) fields. Top funders were ranked by number of publications funded, and temporal trends were analyzed. Co-authorship networks at the country and institutional levels were constructed using the biblioNetwork function in Bibliometrix. Network metrics included degree centrality and betweenness centrality. Both Author Keywords (DE) and Keywords Plus (ID) were extracted and analyzed for co-occurrence.

Network metrics were calculated as follows:

Degree centrality: The raw degree *k_i_* of node *i* is the number of unique collaborators (countries or institutions) connected to node *i*. Normalized degree centrality was calculated as:

$\mathrm{Degree}_{\mathrm{norm}}(i) = \frac{k_i}{n - 1}$

Where *n* is the total number of nodes in the network. For the country-level network, n = 87 (46 SSA countries + 41 non-SSA countries with ≥1 publication). For the institutional network, n = 1,247 (unique institutions with ≥1 publication).

Betweenness centrality: The raw betweenness centrality *b_i_*​ of node *i* is the number of shortest paths between other nodes that pass through node *i*. Normalized betweenness centrality was calculated as:

$\mathrm{Betweenness}_{\mathrm{norm}}(i) = \frac{2b_i}{(n - 1)(n - 2)}$

This normalization scales betweenness to the range {0,1}, where 0 indicates no bridging role, and 1 indicates that all shortest paths pass through the node.

Keywords Plus are algorithmically generated terms from cited references and often capture core concepts more consistently across publications, while Author Keywords reflect authors' own conceptual framing and may show more variation [24]. For co-occurrence analysis, Keywords Plus was used to minimize variability and ensure comparability across publications. For trend analysis (emerging topics), Author Keywords were used to capture authors' evolving conceptualizations of the field. In cases where both fields were available, we created a combined keyword set after normalizing synonyms (e.g., "GBS" and "Group B Streptococcus" were merged).

Research Equity Analysis Methods

To assess research equity, we calculated the following indicators:

Research-to-mortality ratio (RMR): SSA bears approximately 40% of the global neonatal sepsis mortality burden (estimated 98,400 deaths annually, 95% uncertainty interval (UI): 87,200-110,100) but contributed only 4.2% of global neonatal sepsis research output (1,247 of 29,690 global publications) over the study period. The RMR for SSA was 0.38 articles per 1,000 neonatal sepsis deaths (95% UI: 0.34-0.43), compared to 12.7 (95% UI: 11.9-13.5) for North America and 9.4 (95% UI: 8.8-10.0) for Europe. This represents a 33-fold disparity between SSA and North America (ratio: 12.7/0.38 = 33.4) and a 25-fold disparity between SSA and Europe (ratio: 9.4/0.38 = 24.7).

Gross domestic product-adjusted publication rate (GAPR): When adjusted for gross domestic product (GDP), SSA produced 0.12 articles per $1 billion GDP (95% UI: 0.11-0.13), compared to 3.45 (95% UI: 3.32-3.58) for North America and 2.87 (95% UI: 2.75-2.99) for Europe. This indicates a 28.8-fold disparity in research investment relative to economic capacity between SSA and North America (ratio: 3.45/0.12 = 28.8) and a 23.9-fold disparity between SSA and Europe (ratio: 2.87/0.12 = 23.9).

Disparity index (DI): The disparity index was calculated as follows:

$\mathrm{DI}=\frac{\left(\mathrm{SSA\ publications}/\mathrm{Global\ publications}\right)}{\left(\mathrm{SSA\ mortality}/\mathrm{Global\ mortality}\right)}$

$\mathrm{DI}=\displaystyle \frac{(98,\!400/246,\!000)}{(1,\!247/29,\!690)}$

$\displaystyle = \frac{0.400}{0.042}$

*=0.105*

A DI of 1.0 would indicate proportional research investment relative to mortality burden. Our DI of 0.105 indicates that SSA's research output is approximately 10.5% of what would be expected based on its mortality burden - a 90% deficit in research investment relative to disease burden. The 95% uncertainty interval for the DI, derived from Global Burden of Disease (GBD) 2021 mortality uncertainty ranges, was 0.093-0.118.

Ethical Considerations

This study involved no human or animal subjects and therefore did not require ethical approval. All data were publicly available through Scopus and Web of Science.

Results

**Figure 1:**
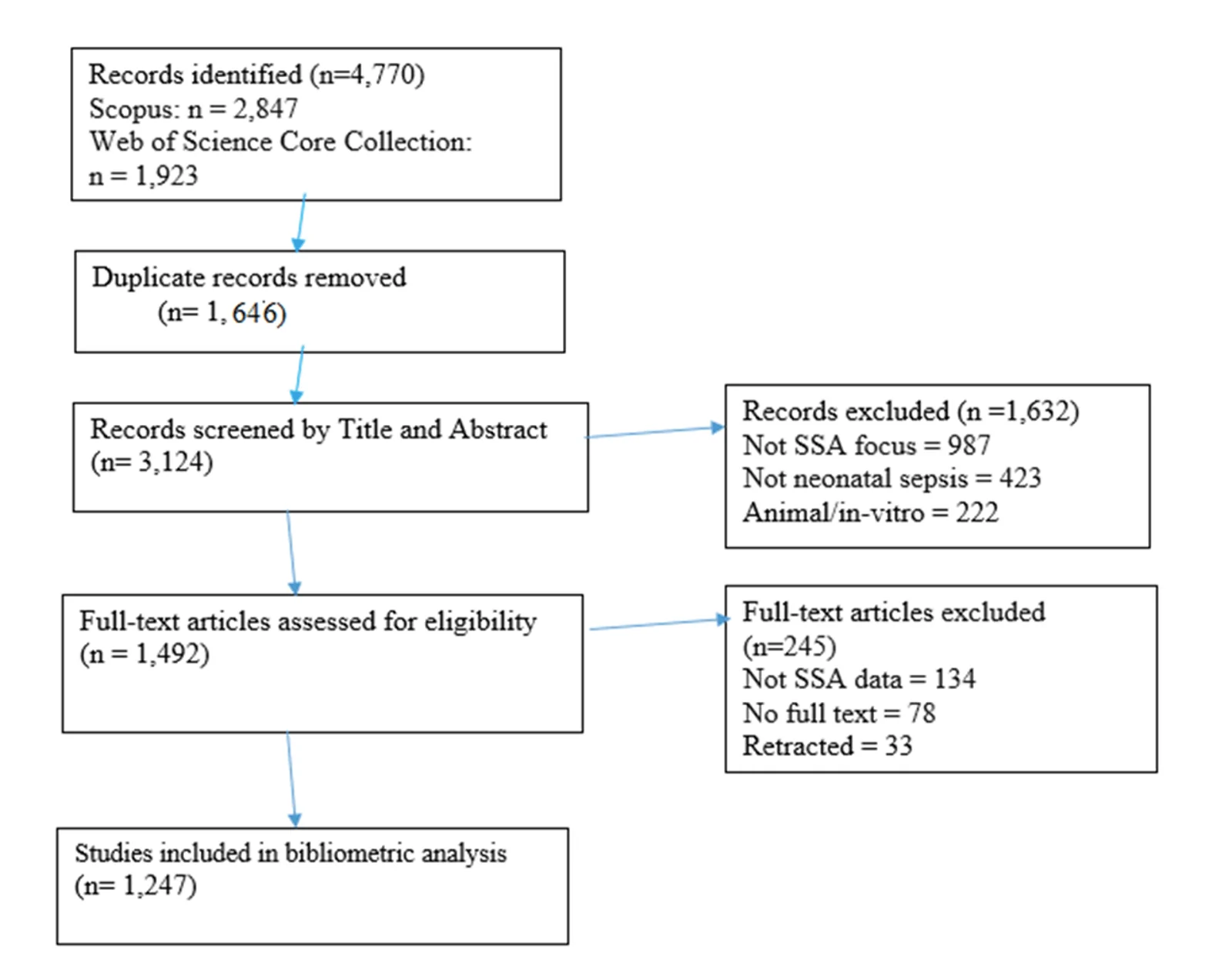
PRISMA 2020 flow diagram for study selection. A total of 4,770 records were identified from Scopus and Web of Science Core Collection. After deduplication and screening, 1,492 full-text articles were assessed for eligibility, and 1,247 studies were included in the final bibliometric analysis. Abbreviations: PRISMA, Preferred Reporting Items for Systematic Reviews and Meta-Analyses; SSA, Sub-Saharan Africa.

A total of 1,247 publications were included in the final analysis. The PRISMA flow diagram for study identification and screening is presented in Figure 1.

Publication Trends

**Figure 2:**
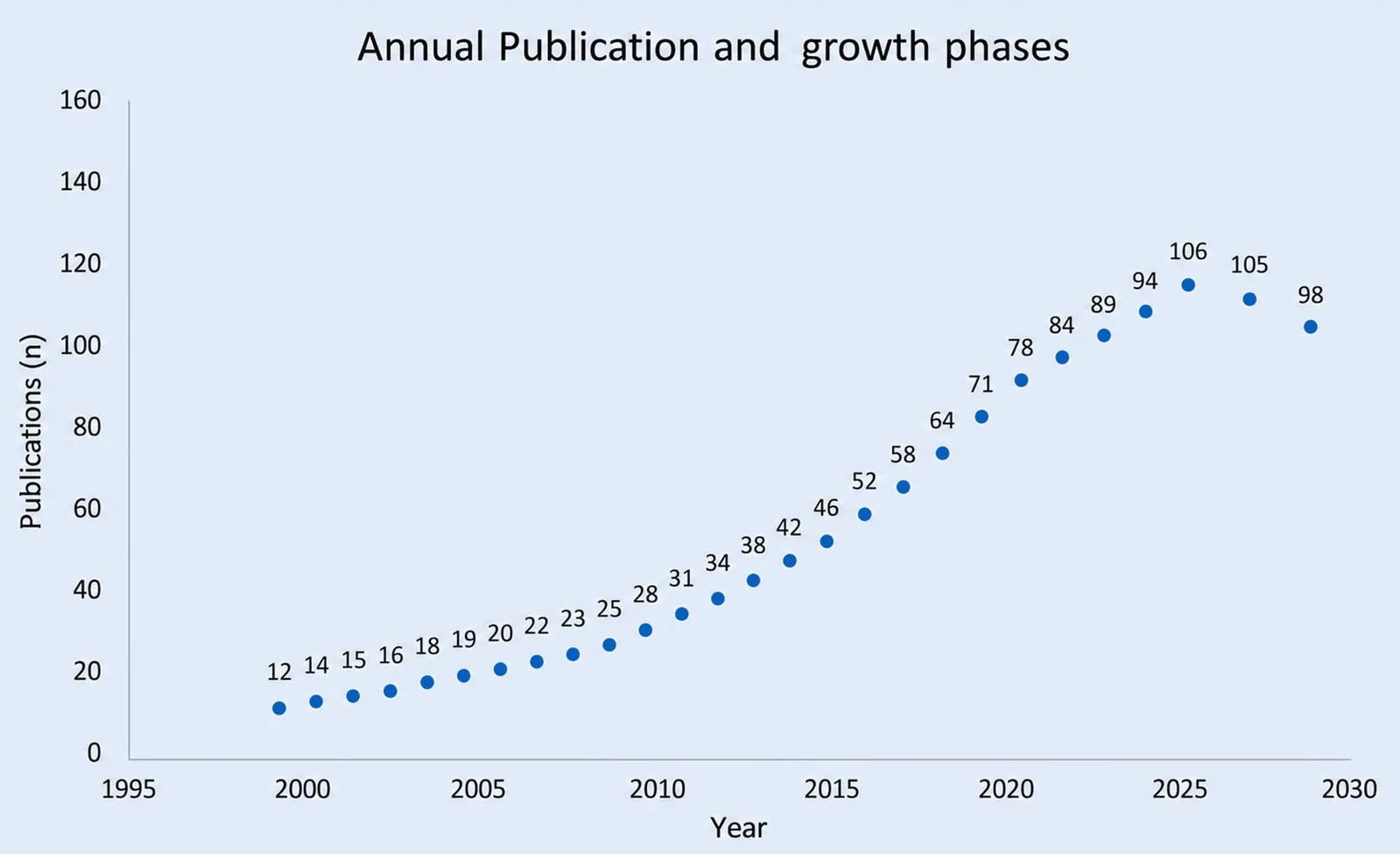
Annual publication trends in neonatal sepsis research in Sub-Saharan Africa (2000-2025). Annual publication trends from 2000 to 2025, illustrating three growth phases: slow growth (2000-2009), moderate growth (2010-2019), and rapid growth (2020-2025). The line graph shows the annual number of publications, and the shaded backgrounds denote the corresponding growth phases.

Figure 2 presents the annual publication trends for neonatal sepsis research in SSA from 2000 to 2025. A total of 1,247 publications were included in the final analysis. Annual output increased from 12 articles in 2000 to 98 articles in 2025, with an average annual growth rate of 8.2% (95% CI: 7.1-9.3%). The growth trajectory showed three distinct phases: a slow growth period (2000-2009; average 18.4 articles/year), a moderate growth period (2010-2019; average 42.7 articles/year), and a rapid growth period (2020-2025; average 87.3 articles/year). The most productive year was 2024 with 106 publications, followed by a slight decline to 98 publications in 2025 (Figure 2). Total citations for all included publications were 28,456, with an average of 22.8 citations per article. The h-index for the entire dataset was 72.

Geographic Productivity

Table 1 presents the top 10 most productive countries in SSA neonatal sepsis research. South Africa, Nigeria, and Kenya together contributed 54.2% of total SSA publications. Francophone African countries (e.g., Senegal, Côte d'Ivoire, and Burkina Faso) and conflict-affected countries (e.g., Chad, Central African Republic, and South Sudan) had notably lower publication outputs, collectively representing less than 5% of total publications. However, this finding should be interpreted cautiously, as our English-language restriction and the underindexing of Francophone journals may have systematically underestimated research from these countries.

**Table 1:** Top 10 most productive countries in Sub-Saharan Africa (SSA) neonatal sepsis research (2000-2025). Top 10 contributing countries to publications on the study topic (2000-2025), ranked by number of articles. The table summarizes publication output, proportion of total publications, citation impact, average citations per article, and the multiple-country publication (MCP) ratio, an indicator of international research collaboration. * MCP = Multiple-country publication ratio. Higher values indicate greater international collaboration.

Rank	Country	Articles (n)	% of total	Total citations	Average citations/article	MCP ratio*
1	South Africa	353	28.3	8,124	23.0	0.38
2	Nigeria	196	15.7	3,892	19.9	0.29
3	Kenya	127	10.2	2,934	23.1	0.44
4	Uganda	89	7.1	1,976	22.2	0.41
5	Ethiopia	76	6.1	1,432	18.8	0.33
6	Ghana	62	5.0	1,234	19.9	0.37
7	Tanzania	54	4.3	987	18.3	0.40
8	Malawi	48	3.9	876	18.3	0.42
9	Zambia	41	3.3	712	17.4	0.35
10	USA (non-SSA)	37	3.0	1,234	33.4	1.00

Table 2 ranks the top 10 most productive institutions. The University of Cape Town (South Africa) led with 94 articles and an H-index of 31, followed by Makerere University (Uganda) and the University of Nairobi (Kenya). The University of Witwatersrand (South Africa), despite ranking fourth, demonstrated the second-highest research impact (H-index: 28). The University of Ibadan (Nigeria) was the sole West African representative in the top 10.

**Table 2:** Top 10 most productive institutions (2000-2025). Top 10 contributing institutions to publications on the study topic (2000-2025), ranked by number of articles. The table summarizes institutional research output, total citations, and H-index as indicators of scientific productivity and research impact. Abbreviation: H-index, Hirsch index.

Rank	Institution	Country	Articles (n)	Total citations	H-index
1	University of Cape Town	South Africa	94	2,456	31
2	Makerere University	Uganda	67	1,543	24
3	University of Nairobi	Kenya	58	1,234	22
4	University of Witwatersrand	South Africa	56	1,876	28
5	University of Ibadan	Nigeria	48	987	19
6	University of Ghana	Ghana	42	876	17
7	Muhimbili University of Health and Allied Sciences	Tanzania	39	712	16
8	Addis Ababa University	Ethiopia	37	654	15
9	University of Malawi	Malawi	34	543	14
10	University of Zambia	Zambia	31	432	12

The ranking reveals a predominance of East and Southern African institutions, highlighting a research-to-burden gap in West Africa. Nigeria, despite being the most populous country in SSA and bearing a substantial proportion of the region's neonatal mortality burden [1,2], ranks fifth with only 48 publications - a disproportionately low output relative to its disease burden.

Funding Sources

Table 3 presents the top 10 funding agencies for SSA neonatal sepsis research from 2000 to 2025. Funding data were available for 876 publications (70.2% of total). HIC-based agencies supported 82.3% of funded studies, while African national funders provided only 6.5%.

**Table 3:** Top 10 funding agencies (2000-2025). Top 10 funding agencies supporting publications on the study topic (2000-2025), ranked by the number of funded articles. The table summarizes the funding agency, country of origin, number of funded articles, and percentage contribution to all funded publications. * Percentages represent the proportion of publications with available funding data. Abbreviations: NIH, National Institutes of Health; MRC, Medical Research Council; NRF, National Research Foundation; WHO, World Health Organization; EDCTP, European & Developing Countries Clinical Trials Partnership; AAS, African Academy of Sciences.

Rank	Funding agency	Country of agency	Articles funded (n)	% of total funded articles*
1	National Institutes of Health (NIH)	USA	137	15.6
2	Wellcome Trust	UK	108	12.3
3	Bill & Melinda Gates Foundation	USA	86	9.8
4	European Commission	EU	67	7.6
5	Medical Research Council (MRC) UK	UK	54	6.2
6	National Research Foundation (NRF) South Africa	South Africa	32	3.7
7	World Health Organization (WHO)	Switzerland	28	3.2
8	European & Developing Countries Clinical Trials Partnership (EDCTP)	EU	24	2.7
9	Fogarty International Center (NIH)	USA	19	2.2
10	African Academy of Sciences (AAS)	Kenya	16	1.8

Collaboration Networks

Table 4 presents the country collaboration network for SSA neonatal sepsis research. The collaboration network revealed strong North-South links, with the USA (link strength = 124), UK (link strength = 98), and the Netherlands (link strength = 67) being the most connected non-SSA countries. Among SSA countries, South Africa (link strength = 87), Kenya (link strength = 65), and Uganda (link strength = 54) showed the highest centrality. South-South collaborations were limited, accounting for only 12.4% of all international collaborations. The most common South-South collaborations were between South Africa and Nigeria (n = 18), Kenya and Uganda (n = 14), and South Africa and Kenya (n = 12). The MCP ratio for SSA countries averaged 0.34 (range: 0.29-0.44), indicating that only about one-third of publications involved international collaboration. MCP ratios were higher for countries with established research partnerships (Kenya: 0.44; Uganda: 0.41) and lower for countries with limited research infrastructure (Nigeria: 0.29; Ethiopia: 0.33).

**Table 4:** Country collaboration network metrics (2000-2025). Link strength: total weighted collaboration volume. Degree centrality: proportion of direct partners (range: 0-1). Between centrality: frequency as a network bridge (range: 0-1). Top partners: strongest collaborative ties. Countries are grouped as non‑SSA (non‑Sub‑Saharan Africa: United States of America (USA), United Kingdom (UK), Netherlands, Germany, Canada) and SSA (Sub‑Saharan Africa: South Africa, Kenya, Uganda, Nigeria, Ghana, Tanzania, Malawi, Ethiopia, Zambia). All centrality values are normalized.

Country	Link strength (Total)	Degree centrality	Between centrality	Top collaboration partners
Non-SSA countries				
USA	124	0.87	0.42	UK, South Africa, Kenya, Uganda
UK	98	0.82	0.38	USA, South Africa, Kenya, Nigeria
Netherlands	67	0.71	0.31	UK, South Africa, Uganda
Germany	54	0.65	0.27	UK, USA, Tanzania
Canada	43	0.58	0.23	USA, UK, Kenya
SSA countries				
South Africa	87	0.76	0.35	USA, UK, Netherlands, Nigeria, Kenya
Kenya	65	0.69	0.29	USA, UK, South Africa, Uganda
Uganda	54	0.63	0.25	USA, UK, Kenya, South Africa
Nigeria	42	0.56	0.21	UK, USA, South Africa, Ghana
Ghana	34	0.49	0.18	UK, USA, Nigeria, South Africa
Tanzania	28	0.43	0.15	UK, USA, South Africa, Kenya
Malawi	23	0.38	0.12	USA, UK, South Africa, Kenya
Ethiopia	19	0.34	0.10	USA, UK, South Africa
Zambia	16	0.30	0.08	USA, UK, South Africa

Table 5 reveals the institutional collaboration network (2000-2025). Three main institutional clusters emerged: a Southern African cluster centered on the University of Cape Town and the University of Witwatersrand; an East African cluster centered on Makerere University and the University of Nairobi; and a West African cluster centered on the University of Ibadan. These clusters were primarily linked through partnerships with HIC institutions rather than direct collaboration with each other.

**Table 5:** Institutional collaboration clusters (2000-2025). Cluster: research collaboration grouping by geographic region (Southern African, East African, West African) or cross‑regional networks. Region: Sub‑Saharan African (SSA) area covered. Core institutions: lead SSA research organizations within each cluster. Key HIC partners: main collaborating institutions from high‑income countries (HIC), including the United States of America (USA) and the United Kingdom (UK). Collaboration density: network density score (range: 0-1), where higher values indicate a greater proportion of possible collaborative ties realized within the cluster.

Cluster	Region	Core institutions	Key HIC partners	Collaboration density
Southern African	Southern Africa	University of Cape Town, University of Witwatersrand, Stellenbosch University	London School of Hygiene & Tropical Medicine, University of Oxford, CDC	High (0.67)
East African	East Africa	Makerere University, University of Nairobi, Muhimbili University, Kenya Medical Research Institute (KEMRI)	University of Washington, University of Liverpool, Karolinska Institutet	Moderate (0.52)
West African	West Africa	University of Ibadan, University of Ghana, University of Lagos	London School of Hygiene & Tropical Medicine, University of Birmingham	Moderate (0.48)
Cross-regional	Multiple	Various SSA institutions	Various HIC institutions	Low (0.23)

Keyword and Theme Analysis

Table 6 presents the dominant research themes (keywords appearing in ≥5% of publications). Keyword co-occurrence reveals that pathogen microbiology (18.4%) and antibiotic resistance (14.2%) dominate the literature. The most frequently isolated pathogens are Group B *Streptococcus*, *Klebsiella pneumoniae*, and *Staphylococcus aureus*. Risk factors (11.8%), including preterm birth, low birth weight, and maternal fever, and diagnosis (9.6%), including blood culture and molecular methods, also feature prominently.

**Table 6:** Dominant research themes in Sub-Saharan Africa (SSA) neonatal sepsis research (2000-2025). Major research themes and representative keywords in neonatal sepsis literature (2000-2025). This table summarizes the most frequently occurring research themes identified through keyword co-occurrence analysis, with their relative frequencies and representative keywords. Abbreviations: AMR, antimicrobial resistance; MRSA, methicillin-resistant Staphylococcus aureus; ESBL, extended-spectrum beta-lactamase; MDRO, multidrug-resistant organisms; CRP, C-reactive protein; PCT, procalcitonin; PCR, polymerase chain reaction; CBC, complete blood count; MODS, multiple organ dysfunction syndrome; NICU, neonatal intensive care unit; EOS, early-onset sepsis; LOS, late-onset sepsis.

Theme	Frequency (%)	Representative keywords
Microbiology/pathogens	18.4%	Streptococcus agalactiae (Group B Streptococcus), Klebsiella pneumoniae, Staphylococcus aureus, Escherichia coli, Pseudomonas aeruginosa, Enterobacter spp., Coagulase-negative staphylococci
Antibiotic resistance	14.2%	Antimicrobial resistance (AMR), methicillin-resistant Staphylococcus aureus (MRSA), extended-spectrum beta-lactamase (ESBL), carbapenem resistance, multidrug-resistant organisms (MDRO), antimicrobial susceptibility testing
Risk factors	11.8%	Preterm birth, low birth weight, maternal fever, prolonged rupture of membranes, intrapartum antibiotic prophylaxis, maternal chorioamnionitis, neonatal asphyxia, home delivery, delayed seeking of care
Diagnosis	9.6%	Blood culture, C-reactive protein (CRP), procalcitonin (PCT), molecular diagnostics, polymerase chain reaction (PCR), rapid diagnostic tests, point-of-care testing, complete blood count (CBC)
Mortality/outcomes	8.4%	Neonatal mortality, case fatality rate, septic shock, multiple organ dysfunction syndrome (MODS), neonatal intensive care unit (NICU) outcomes, long-term neurodevelopmental outcomes
Epidemiology	6.3%	Disease burden, incidence rates, prevalence, case definitions, population-based studies, surveillance, early-onset sepsis (EOS), late-onset sepsis (LOS)
Antimicrobial therapy	5.7%	Empirical antibiotics, antibiotic stewardship, vancomycin, gentamicin, amikacin, third-generation cephalosporins, carbapenems, duration of therapy, therapeutic drug monitoring

Emerging Research Topics in Neonatal Sepsis

Table 7 highlights emerging research topics in neonatal sepsis, comparing pre-2015 and post-2015 publication frequencies. Antimicrobial resistance (AMR) surveillance increased from 1.2% to 5.6% (367% growth), driven by the global AMR action plan. Sepsis bundles and quality improvement rose from 0.8% to 3.9% (388%), influenced by WHO Sepsis Resolutions. Machine learning/AI showed the highest growth (950%, from 0.2% to 2.1%), reflecting advances in digital health and predictive analytics. Point-of-care diagnostics grew from 1.0% to 4.2% (320%), driven by molecular technologies like GeneXpert. Implementation science increased from 0.5% to 2.1% (320%), addressing the know-do gap, while telemedicine rose from 0.3% to 1.9% (533%), accelerated by the COVID-19 pandemic. These trends indicate a shift toward technology-driven and implementation-focused research to combat neonatal sepsis in SSA.

**Table 7:** Emerging research topics in neonatal sepsis literature pre and post 2015. The table compares the prevalence of emerging research topics in the pre-2015 and post-2015 periods, showing percentage growth and key drivers of their emergence. * Growth rate calculated as: [(Post-2015 % - Pre-2015 %) / Pre-2015 %] × 100. Abbreviations: AMR, antimicrobial resistance; SSA, Sub-Saharan Africa; WHO, World Health Organization; mHealth, mobile health; EMR, electronic medical record; GeneXpert, GeneXpert molecular diagnostic platform; UHC, universal health coverage; COVID-19, coronavirus disease 2019.

Emerging topic	Baseline (Pre-2015)	Post-2015	Growth rate*	Rationale for emergence
Antimicrobial resistance surveillance	1.2%	5.6%	367%	Global AMR action plan (2015); rising resistance rates in SSA; need for local data to guide empirical therapy
Sepsis bundles and quality improvement	0.8%	3.9%	388%	WHO Sepsis Resolutions (2017); Surviving Sepsis Campaign; focus on reducing preventable deaths
Machine learning/artificial intelligence	0.2%	2.1%	950%	Advances in digital health; mobile health (mHealth); predictive algorithms using EMR data
Point-of-care diagnostics	1.0%	4.2%	320%	WHO ASSURED criteria; need for affordable, rapid tests; molecular diagnostics (GeneXpert, BioFire)
Implementation science	0.5%	2.1%	320%	Recognition of "know-do" gap; need for context-specific implementation frameworks; global focus on UHC
Telemedicine/remote care	0.3%	1.9%	533%	COVID-19 pandemic; expansion of telemedicine; need for specialist consultation in remote settings

Under-Researched Areas in Neonatal Sepsis

Table 8 identifies under-researched areas in neonatal sepsis, ranked by priority rating. Implementation science (2.1%), health systems research (1.8%), and prevention strategies (2.3%) were all rated as critically under-researched (Priority 5), with gaps including strategies for integrating evidence-based interventions into routine practice, supply chain management, workforce training, and community-based prevention such as maternal immunization and chlorhexidine cord care. Community-based interventions (1.5%) and health economics (0.9%) were rated as high priority (Priority 4), with gaps in community case management, health worker training, cost-effectiveness analyses, and economic burden assessment. These findings highlight the urgent need for research on how to effectively deliver, sustain, and finance interventions in resource-limited settings to reduce the burden of neonatal sepsis in the region.

**Table 8:** Under-researched areas and research priorities in neonatal sepsis literature (2000-2025). The table highlights under-researched thematic areas, their relative frequencies, identified research gaps, and priority ratings for future investigation. Priority ratings range from 1 (lowest) to 5 (critical) and are based on the potential impact of addressing the identified research gaps. * Priority rating: 1 = low; 2 = moderate-low; 3 = moderate; 4 = high; 5 = critical.

Under-researched area	Frequency (%)	Research gap description	Priority rating*
Implementation science	2.1%	Strategies to integrate evidence-based interventions into routine practice; frameworks for context-specific adaptation	5 (Critical)
Health systems research	1.8%	Supply chain management; health workforce training; facility readiness; referral systems; cost-effectiveness	5 (Critical)
Prevention strategies	2.3%	Community-based prevention; maternal immunization; chlorhexidine cord care; hand hygiene; infection prevention and control	5 (Critical)
Community-based interventions	1.5%	Community case management; community health worker training; home-based newborn care; health promotion	4 (High)
Health economics	0.9%	Cost-effectiveness analyses; economic burden of neonatal sepsis; financial protection; return on investment	4 (High)

Temporal Trends in Neonatal Sepsis Research Themes

Table 9 presents temporal trends in neonatal sepsis research themes across three periods. "Microbiology/pathogens" declined from 22.1% to 16.3%, while "Mortality/outcomes" decreased from 10.2% to 7.1%, indicating declining research focus on pathogen identification and mortality. Conversely, "Antibiotic resistance" increased from 8.7% to 19.7%, reflecting growing concern over AMR. "Diagnosis" rose from 7.8% to 11.2%, driven by advances in molecular diagnostics. The most notable growth occurred in "Implementation science" (0.2% to 5.1%), "Health systems research" (0.1% to 4.8%), and "Point-of-care diagnostics" (0.3% to 6.5%), highlighting a paradigm shift toward translational research, health systems strengthening, and accessible diagnostic technologies. "Risk factors" remained relatively stable (14.5% to 10.2%). These trends reflect the evolving research priorities in response to the persistent burden of neonatal sepsis in SSA.

**Table 9:** Temporal trends in major research themes in neonatal sepsis literature (2000-2025). The table illustrates changes in the relative frequency of major research themes across three time periods (2000-2009, 2010-2019, and 2020-2025). Arrows indicate overall trends over time (↑ rising, ↓ declining, ↔ stable). Topics such as antibiotic resistance, point-of-care diagnostics, implementation science, and health systems research showed increasing prominence, whereas microbiology/pathogens and mortality/outcomes declined proportionally.

Theme	2000-2009 (%)	2010-2019 (%)	2020-2025 (%)	Trend direction
Microbiology/pathogens	22.1%	18.9%	16.3%	↓ Declining
Antibiotic resistance	8.7%	12.4%	19.7%	↑ Rising
Risk factors	14.5%	12.1%	10.2%	↔ Stable
Diagnosis	7.8%	8.9%	11.2%	↑ Rising
Mortality/outcomes	10.2%	8.7%	7.1%	↓ Declining
Implementation science	0.2%	0.8%	5.1%	↑ Rising
Health systems research	0.1%	0.7%	4.8%	↑ Rising
Point-of-care diagnostics	0.3%	1.2%	6.5%	↑ Rising

Research Equity Analysis

SSA bears approximately 40% of the global neonatal sepsis mortality burden but contributed only 4.2% of global neonatal sepsis research output over the study period. The research-to-mortality ratio (articles per 1,000 neonatal sepsis deaths) was 0.38 for SSA compared to 12.7 for North America and 9.4 for Europe. When adjusted for GDP, SSA produced 0.12 articles per $1 billion GDP compared to 3.45 for North America and 2.87 for Europe, indicating a 28-fold disparity in research investment relative to economic capacity. The disparity index (DI) was 0.105, indicating that SSA's research output is approximately 10% of what would be expected based on its mortality burden.

Discussion

Key Findings in the Context of Disease Burden

Over 25 years, we identified 1,247 publications on neonatal sepsis with SSA focus, with exponential growth post 2010. Annual output increased from 12 articles in 2000 to a peak of 106 in 2024, followed by a slight decline to 98 in 2025, representing an average annual growth rate of 8.2%. However, SSA's contribution remains disproportionately low relative to its mortality burden, which accounts for approximately 40% of global neonatal deaths [1,2]. This disparity highlights a research equity gap that may limit evidence-based interventions tailored to SSA contexts [25-28].

The growth trajectory observed parallels global trends in neonatal sepsis research but lags behind the rapid growth seen in infectious disease research more broadly in SSA [29,30]. The accelerated growth post 2015 likely reflects the influence of the Sustainable Development Goals (SDGs) and initiatives such as the Global Action Plan to Combat Antimicrobial Resistance [31]. However, the persistent gap between disease burden and research output suggests that these initiatives have not yet fully translated into equitable research investment.

The slight decline from 106 publications in 2024 to 98 in 2025 warrants attention. While the overall trajectory remains upward, this interruption may reflect several factors. First, the peak in 2024 may represent the culmination of multiple major grant cycles (typically five years) initiated between 2019 and 2020, with publications declining as these projects conclude. Second, the COVID-19 pandemic may have created a "catch-up" effect in 2023-2024 that subsequently normalized. Third, it is possible that 2025 publications are partially underrepresented in Scopus and Web of Science due to indexing delays, a common limitation of bibliometric analyses that include the most recent year [32]. Nevertheless, this observation highlights the fragility of research output in SSA and underscores the need for sustained, predictable funding mechanisms rather than boom-and-bust cycles driven by short-term donor priorities.

Geographic Productivity and Institutional Patterns

The dominance of South Africa, Nigeria, and Kenya in neonatal sepsis research reflects established research infrastructure, university systems, and partnerships with HICs [33,34]. South Africa's leadership is consistent with its position as the most research-intensive country in SSA, with well-established neonatal intensive care networks and infectious disease research programs [35].

The underrepresentation of Francophone African countries and conflict-affected states (e.g., Chad, Central African Republic, and Niger) is concerning given their high neonatal mortality rates [36]. This pattern mirrors known inequities in African research capacity and highlights the need for targeted investment in fragile and conflict-affected settings [37,38]. Language barriers and limited access to international databases may further marginalize Francophone research output [39].

Top institutions, including the University of Cape Town, Makerere University, and the University of Nairobi, serve as regional hubs for neonatal sepsis research. Their productivity is closely linked to partnerships with HIC institutions through programs such as the NIH's Medical Education Partnership Initiative (MEPI) and the Wellcome Trust's African institutions [40,41]. These partnerships have been instrumental in building research capacity, but their concentration in a few institutions raises concerns about regional disparities. This geographic concentration is particularly striking when considering West Africa. Nigeria, despite being the most populous country in SSA and bearing a substantial proportion of the region's neonatal mortality burden [1,2], has only one institution, i.e., the University of Ibadan, in the top 10, ranking fifth with 48 publications. This contrasts sharply with the institutional density in East and Southern Africa, where multiple institutions from Kenya, Uganda, Tanzania, Malawi, Zambia, and South Africa appear in the rankings. The underrepresentation of Nigerian and other West African institutions suggests that research investment is not aligned with the regional distribution of neonatal sepsis mortality, raising critical questions about research equity and priority-setting.

Funding Patterns and Research Equity

Our funding analysis reveals near-total dependence on HIC agencies, with NIH, Wellcome Trust, and Bill & Melinda Gates Foundation accounting for 37.7% of all funded studies. African national funders contributed less than 6.5% of funding, with the National Research Foundation (South Africa) and African Academy of Sciences being the largest African funders. This pattern raises concerns about sustainability, local ownership, and the alignment of research agendas with local priorities [42].

The dominance of HIC funding may result in "helicopter research" - a term used to describe research conducted in low-income settings where external researchers design the study, collect data, and publish findings with limited local involvement, capacity building, or benefit to the host community [43,44]. This pattern has been documented across multiple health research domains in SSA and is associated with misaligned research priorities, limited local ownership, and reduced translation to practice [45].

Funding trends showed a slight increase in African-funded research post 2015, likely reflecting the SDG era and initiatives such as the African Union's Agenda 2063 [46]. However, this increase remains modest, and substantial additional investment from African governments and regional bodies is needed to shift the balance of research funding.

Collaboration Networks: North-South vs. South-South

Country collaboration networks revealed strong North-South links, with the USA, UK, and Netherlands being the most connected partners. These partnerships are associated with higher citation impact (as reflected in higher MCP ratios and average citations per article), consistent with previous findings on the advantages of international collaboration [47].

South-South collaboration, however, remains limited. Only 12.4% of international collaborations occurred between SSA countries, and MCP ratios for SSA countries averaged 0.34, indicating that only one-third of publications involved international collaboration. This paucity of South-South networks limits regional knowledge sharing and reduces the efficiency of research capacity building [45].

The three institutional clusters (Southern, East, and West Africa) are primarily linked through HIC partners rather than direct collaboration with each other. This "hub-and-spoke" model of collaboration reduces opportunities for SSA institutions to learn from each other's experiences and share contextually relevant solutions [48]. We use the terms "hub-and-spoke" and "hub-to-hub" to describe two distinct collaboration models. In the hub-and-spoke model, SSA institutions primarily collaborate with HIC "hub" institutions, with limited direct collaboration between SSA institutions. This model is evident in our network analysis, where the three SSA institutional clusters are linked primarily through HIC partners. In the hub-to-hub model, SSA institutions directly collaborate with each other, creating a more distributed network. This model, which we recommend, would facilitate regional knowledge sharing, reduce dependency on external partners, and align research priorities across the region [49].

Research Themes and Knowledge Gaps

Dominant themes identified through keyword analysis (microbiology, antibiotic resistance, and risk factors) reflect the clinical focus of neonatal sepsis research in SSA. The emphasis on microbiology is appropriate, given the high burden of AMR in SSA and the need for local surveillance data to guide empirical therapy [50,51].

Emerging topics post 2015, including AMR surveillance, sepsis bundles, and machine learning, indicate a shift toward more sophisticated research approaches. The growing interest in point-of-care diagnostics reflects the recognition that resource-appropriate tools are needed to address the diagnostic gap in low-resource settings [52].

However, several areas remain under-researched, including implementation science, health systems research, prevention strategies, community-based interventions, and health economics. These gaps are concerning because evidence-based implementation of proven interventions, such as chlorhexidine cord care, Kangaroo Mother Care, and hand hygiene, is as important as identifying new therapeutic approaches [53,54]. The lack of implementation science research may contribute to the "know-do gap" where effective interventions fail to reach newborns due to implementation barriers [55].

To illustrate these research gaps, we provide concrete examples of under-researched areas. In implementation science, key priorities include: (1) evaluating strategies to integrate neonatal sepsis screening into routine antenatal and postnatal care at primary health facilities; (2) identifying barriers to uptake of chlorhexidine cord care and Kangaroo Mother Care in different SSA settings; and (3) developing context-specific implementation frameworks for sepsis quality improvement bundles. In health systems research, priorities include: (1) assessing the cost-effectiveness of different sepsis diagnostic pathways; (2) evaluating the impact of task-shifting for neonatal sepsis management; and (3) analyzing supply chain barriers to antibiotic availability at peripheral health facilities. These examples highlight the translational research needed to bridge the gap between evidence and practice.

Practical Guidance for SSA Researchers

Researchers in SSA should actively seek partnerships with institutions in neighboring countries with similar burden profiles. Existing consortia such as the African Neonatal Sepsis Network offer platforms for regional collaboration [56]. They should consider integrating implementation outcomes into clinical studies. Frameworks such as RE-AIM or the Consolidated Framework for Implementation Research (CFIR) can guide study design [57]. To increase visibility and ensure inclusion in bibliometric databases, consider publishing in journals indexed in Scopus and WoS, while also contributing to regional journals that serve local audiences. While HIC funding is important, researchers should actively seek grants from African funding bodies such as the African Academy of Sciences, national research councils, and the European & Developing Countries Clinical Trials Partnership (EDCTP) to strengthen local research ownership [58]. Furthermore, accurate reporting of funding sources and institutional affiliations in publications is essential for future bibliometric analyses and transparency.

Implications for Sustainable Development Goals

Our findings have direct implications for the SDGs. SDG 3.2 aims to end preventable newborn deaths, with a target of reducing neonatal mortality to ≤12 per 1,000 live births by 2030 [59]. Achieving this target in SSA requires context-specific evidence on neonatal sepsis epidemiology, prevention, and treatment, yet our findings reveal persistent gaps in research aligned with local priorities. Furthermore, SDG 17.6 calls for enhanced knowledge sharing and collaboration through North-South and South-South partnerships [60]. Our collaboration network analysis shows that while North-South partnerships are strong, South-South collaborations remain limited (12.4% of international collaborations), indicating missed opportunities for regional knowledge sharing that could accelerate progress toward SDG targets.

Implications and Recommendations

Our findings highlight the need for increased funding for African-led research through mechanisms such as the African Academy of Sciences, the EDCTP, and national research councils. Funders should prioritize South-South collaboration and require evidence of local ownership and engagement in research partnerships [61]. SSA research institutions should invest in building regional networks for neonatal sepsis research, including shared biobanks, training programs, and collaborative clinical trials. Institutional partnerships should aim to move from "hub-and-spoke" to "hub-to-hub" models that facilitate direct exchange between SSA institutions [50]. Also, national governments in SSA should increase domestic funding for health research and use bibliometric data to prioritize research investments aligned with local disease burden. The African Union's commitment to allocating 1% of GDP to research and development should be operationalized to support health research in high-burden areas like neonatal sepsis [62]. Research priorities in SSA neonatal sepsis should shift toward implementation science, health systems research, and prevention strategies. In addition, researchers should actively seek South-South collaboration opportunities and ensure that research translates into locally relevant interventions and policies.

Limitations

This study has several limitations. First, Scopus and Web of Science underindex African journals, particularly those not published in English and those indexed only in African Journals OnLine (AJOL). Studies have estimated that up to 30-40% of health research from Francophone Africa is published in regional French-language journals that are not indexed in these databases [63,64]. Similarly, Lusophone African countries such as Angola and Mozambique may have their research outputs captured primarily in Portuguese-language journals not indexed in Scopus or WoS. This selection bias is an inherent trade-off: including PubMed and AJOL would have provided broader geographic coverage, but would have sacrificed the structured funding and affiliation metadata (FU/FO fields) essential for our collaboration network and funding source analyses. We prioritized the latter to address our specific research questions, but readers should interpret geographic productivity metrics, particularly for Francophone and Lusophone countries, with this limitation in mind.

Second, the restriction to English-language publications introduces a systematic bias against Francophone and Lusophone SSA countries. Research published in French-language journals (e.g., Médecine et Santé Tropicales and Bulletin de la Société de Pathologie Exotique) or Portuguese-language journals (e.g., Revista de Saúde Pública de Angola) is excluded. This bias is particularly relevant for countries like Senegal, Côte d'Ivoire, Burkina Faso, Democratic Republic of the Congo, Angola, and Mozambique, which may have substantial scientific outputs in their official languages that are not captured in our analysis.

Third, funding data were incomplete, particularly for publications before 2008 when Scopus began systematically capturing funding information. This may have underestimated African funding sources that are less frequently captured in international databases.

Fourth, bibliometric indicators measure publication output, not research quality or impact on clinical outcomes. High citations do not necessarily translate to changes in clinical practice or reductions in mortality [48].

Fifth, SSA is a diverse region, and aggregating data masks substantial heterogeneity between countries (e.g., South Africa vs. Sahel countries). Future research should disaggregate data by sub-region and country income level.

Finally, our search strategy may have missed studies that focused on neonatal sepsis but did not include specific country names or "Sub-Saharan Africa" in the title, abstract, or keywords. We attempted to mitigate this by including a comprehensive list of all 46 SSA countries in the search string.

## Conclusions

Despite a 25-year increase in output, neonatal sepsis research in SSA remains disproportionately low relative to disease burden and depends heavily on external funding. Strengthening African-led funding mechanisms and fostering South-South collaborations are essential to build sustainable research capacity and align research priorities with local mortality burden. To our knowledge, this is the first 25-year bibliometric analysis exclusively focused on neonatal sepsis research in SSA, providing a baseline for monitoring progress toward research equity. Addressing these gaps through targeted funding, regional collaboration, and implementation science will be critical to reducing neonatal sepsis mortality in SSA and achieving the SDG target for newborn survival.
